# Therapeutic and Pharmaceutical Potential of *Scutellaria baicalensis*-Derived Exosomes for Oily Skin Disorders

**DOI:** 10.3390/antiox14030364

**Published:** 2025-03-19

**Authors:** Guybin Gong, Mihae Yun, Ohhyuk Kwon, Boyong Kim

**Affiliations:** 1 Department of Management of Beauty and Design, College of Design, Hansung University, Seoul 02876, Republic of Korea; 99220203@hansung.ac.kr (G.G.); beauty67@hansung.ac.kr (O.K.); 2 Department of Dental Hygiene, Andong Science College, Andong 36729, Republic of Korea; ymh0710@asc.ac.kr; 3 EVERBIO, 131, Jukhyeon-gil, Gwanghyewon-myeon, Jincheon-gun 27809, Republic of Korea

**Keywords:** acne, fine dust, extracellular vesicle, sebum, dermal lipogenesis

## Abstract

Background: Fine dust exposure worsens oily skin by disrupting lipid metabolism and triggering oxidative inflammation. *Scutellaria baicalensis* extract-induced exosomes (SBEIEs) have shown anti-inflammatory effects by suppressing reactive oxygen species (ROS) and lipid-regulating properties, making them potential therapeutic agents. Methods: Exosomes from fibroblasts treated with SBEIEs and PM10 were tested on macrophages, adipose-derived stem cells (ASCs), and T lymphocytes. ELISA, flow cytometry, and PCR measured cytokines and gene expression. A 10-day clinical trial evaluated skin hydration, oiliness, and inflammation. Results: SBEIEs increased IRF3 (1.6 times) and suppressed PPARγ in ASCs while enhancing lipolysis markers. Sebaceous gland activity (squalene synthase) decreased by 10%. Macrophages showed increased IRF3, IFN-β, and IL-10 (2.1 times). T cells secreted IL-4 and IL-22 (2–2.33 times). Clinically, SBEIEs improved hydration (21%), reduced oiliness (1.6 times), and decreased inflammation (2.2 times). Conclusions: SBEIEs effectively regulate lipid metabolism, cytokines, and immune responses, showing promise to treat oily and inflamed skin caused by fine dust exposure. Further studies are needed for clinical applications.

## 1. Introduction

Adipose-derived stem cells (ASCs) are multipotent stem cells found within adipose tissue that can differentiate into various cell types, including adipocytes, osteocytes, and chondrocytes [1]. Among their many functions, ASCs play a pivotal role in adipogenesis, the process by which preadipocytes mature into adipocytes, contributing to the regulation of body fat mass and overall energy homeostasis [2]. The ability of ASCs to differentiate into adipocytes makes them crucial for tissue regeneration and repair, but this property also implicates them in the pathological expansion of adipose tissue, particularly in the context of obesity and metabolic disorders [3]. Promoting adipocyte differentiation and the consequent increase in adipocyte number due to ASC activity can significantly impact human health. The excessive differentiation of ASCs into adipocytes contributes to adipose tissue expansion, associated with increased fat storage and alterations in adipose tissue function. This can lead to the development of obesity, insulin resistance, type 2 diabetes, and cardiovascular diseases [4]. Enlarged adipose tissue also secretes higher levels of pro-inflammatory cytokines and adipokines, such as TNF-α and IL-6, contributing to chronic low-grade inflammation, further exacerbating metabolic disturbances [5]. The cytokine IL-6 secreted by fibroblasts can promote adipogenesis and lipid accumulation [6,7]. In particular, moderate increases in TNF-α promote lipolysis, whereas excessive secretion induces inflammation [8]. The balance of these cytokines in the local microenvironment can significantly influence the behavior of ASCs and adipose tissue dynamics, contributing to various metabolic and inflammatory conditions [9,10]. Lipogenesis promoted by ASCs plays a significant role in the pathology of skin diseases [11,12]. The interplay between sebaceous glands, adipogenesis, and lipid metabolism affects various skin functions, including barrier function, elasticity, and regeneration [13]. Interferon regulatory factor 3 (IRF3) is a key molecule in lipogenesis and adipocytic differentiation [14]. IRF3 inhibits adipocytic differentiation from ASCs by suppressing peroxisome proliferator-activated receptor gamma (PPARγ). It upregulates interferon β (IFNβ) and interleukin 10 (IL-10) in macrophages for anti-inflammation and the activation of infiltration and M1 polarization in adipose tissues [15,16].

*Scutellaria baicalensis*, commonly known as Baikal skullcap, is a traditional herb widely used in East Asian medicine for its diverse therapeutic effects, primarily attributed to its active flavonoids, including baicalin, baicalein, and wogonin (Li et al., 2019). This herb exhibits potent anti-inflammatory properties by inhibiting key inflammatory pathways, such as NF-κB and MAPK, making it helpful in treating inflammatory conditions [17]. *Scutellaria baicalensis* has substantial antioxidant activities, protecting cells from oxidative stress linked to aging and chronic diseases [18]. It also demonstrates significant anti-cancer effects, inducing apoptosis and inhibiting tumor growth in various cancers [19]. Its antiviral and antibacterial activities target pathogens like hepatitis B and influenza viruses, supporting its role in managing infections [19]. The herb’s neuroprotective effects are beneficial in neurodegenerative diseases, such as Alzheimer’s, reducing neuronal inflammation and oxidative damage [20].

Exosomes are small extracellular vesicles (30–150 nm) released by various cell types, playing a key role in intercellular communication by transporting proteins, lipids, and nucleic acids, including mRNAs and microRNAs [21]. Formed through the endosomal pathway, exosomes are released upon the fusion of multivesicular bodies with the plasma membrane [22]. Initially seen as cellular waste, exosomes are recognized as essential mediators in immune response, cell proliferation, and tissue repair. They are implicated in cancer and neurodegenerative disorders [23]. Recently, exosomes derived from cells treated with natural compounds have shown enhanced therapeutic properties. For example, exosomes from mesenchymal stem cells treated with curcumin exhibit strong anti-inflammatory and antioxidant effects, suggesting potential for treating inflammatory diseases [24]. Similarly, resveratrol-induced exosomes demonstrated anti-cancer effects by modulating signaling pathways and inducing apoptosis [25]. Exosomes offer a novel approach to drug delivery due to their biocompatibility and ability to cross biological barriers, including the blood–brain barrier [26]. Understanding the exosomes’ functions could lead to new therapeutic strategies for various diseases.

Particulate matter (PM) consists of solid particles and liquid droplets in the air, varying in size and origin. PM10, particles with diameters of 10 μm or smaller, can bypass the body’s defenses and penetrate the respiratory system and skin, posing a significant health risk, particularly in urban areas [27]. PM10 originates from natural sources, like dust storms and volcanic eruptions, as well as human activities, such as traffic emissions and industrial processes. Recent studies focused on PM10’s effects on the skin, which serves as the first line of defense against environmental factors. PM10 can adhere to the skin, penetrate through hair follicles, and disrupt the skin barrier, leading to inflammation, oxidative stress, and microbiota alterations [27,28]. These effects are linked to conditions like atopic dermatitis, eczema, acne, and accelerated aging [29]. Oxidative damage from PM10 weakens structural proteins like collagen and elastin, contributing to skin aging and other pathologies [30]. Understanding PM10’s impact on skin health is crucial for developing preventive strategies, as exposure triggers inflammatory pathways and generates reactive oxygen species (ROS), exacerbating skin disorders [31,32]. PM10 exposure triggers oxidative stress by upregulating NADPH oxidase activity, leading to excessive reactive oxygen species (ROS) production [31,32]. This, in turn, activates the NF-κB and MAPK pathways, inducing pro-inflammatory cytokines, such as TNF-α and IL-6 [33]. Additionally, PM10 disrupts lipid homeostasis by modulating mTOR signaling in sebocytes, leading to increased sebum synthesis and lipid accumulation, which exacerbate inflammatory skin conditions [34]. Airborne particulate matter (PM) in Seoul has been shown to damage the skin barrier, exacerbating chronic inflammatory skin conditions such as atopic dermatitis. Heavy metals and carcinogens contained in PM can trigger skin inflammation, leading to adverse effects on skin health [35].

This study isolated exosomes induced by *Scutellaria baicalensis* extract from skin cells and developed a biomaterial for preventing skin diseases caused by particulate matter exposure and various other factors. We hypothesize that SBEIEs will reduce inflammatory cytokine expression and regulate lipid metabolism in a fine dust-exposed skin model by modulating immune responses and suppressing oxidative stress. Based on the pharmacological excellence of exosomes, we further hypothesize that their efficacy as a preventive material against skin disorders could be demonstrated, enabling their application in various product formulations. Both in vitro and in vivo experiments were conducted to validate this idea.

## 2. Materials and Methods

### 2.1. Scutellaria baicalensis Extraction and Cell Culture

Whole *Scutellaria baicalensis* samples dried with infrared rays at 60 °C for 24 h were ground into microparticles (400 mesh). The powder was extracted with distilled water and vacuumed at 0.08 MPa, 70 °C, for two hours. The *Scutellaria baicalensis* extract (SBE) was supplied by EVERBIO Co., Ltd. (Jincheon, Republic of Korea) and filtered through a microporous membrane (0.22 µm, Merck, Darmstadt, Germany). Human fibroblast cells (PCS-201-012, ATCC, Manassas, VA, USA), passage 5, were cultured in Dulbecco’s Modified Eagle Medium (DMEM) in high glucose (Invitrogen, Carlsbad, CA, USA) and supplemented with 10% FBS (Sigma, St. Louis, MO, USA) and 100 U/mL of penicillin (Invitrogen) at 37 °C with 5% humidified CO_2_. Adipose-derived stem cells (ASCs) (Thermo Fisher Scientific, Waltham, MA, USA), passage 5, were cultured in a MesenPRO RS™ Basal Medium, Gibco (Thermo Fisher Scientific) with growth supplement (MesenPRO RS™ Growth Supplement, Thermo Fisher Scientific). Macrophages (KG1, ATCC), passage 7, were cultured under various conditions (CIE, PM10IE, SBEIE, and SBEIE + PM10IE) to evaluate their activity for lipogenic modulation.

### 2.2. Establishment of Treating Doses and Purification of Induced Exosomes

We established treating doses for SBE and fine dust (PM10, ERM-CZ100, Sigma-Aldrich, St. Louis, MO, USA) using the Annexin V-PI apoptosis detection kit (Invitrogen) to analyze the viability of fibroblasts (PCS-201-012, ATCC) using a flow cytometer (BD FACScalibur, BD Biosciences, San Jose, CA, USA) and FlowJo 10.6.1 (BD Biosciences). The induced exosomes (CIEs: control-induced exosomes, SBEIEs: SBE-induced exosomes, PM10IEs: fine dust-induced exosomes) were prepared through fibroblasts exposed to SBE and PM10. To isolate induced exosomes, the cultured fibroblasts were exposed to a control, PM10, and SBE, and the induced exosomes were isolated and purified from the supernatants (10 mL) of the exposed fibroblasts using the exoEasy Maxi Kit (QIAGEN, Hilden, Germany) and CD68 Exo-Flow Capture Kit (System Biosciences, Palo Alto, CA, USA), respectively. To confirm exosome internalization, ASCs were treated with the purified exosomes and then stained with a CD63 antibody (Thermo Fisher Scientific) to visualize intracellular exosomes (S1).

### 2.3. Flowcytometry

After the ASCs were cultured under various conditions (CIE, PM10IE, SBEIE, and SBEIE + PM10IE) for 24 h, the exposed cells were fixed with 2% paraformaldehyde for four hours and treated with 0.02% Tween 20 for five minutes. The same procedures were followed for macrophages (THP-1, ATCC), except for the treatment with 0.02% Tween 20. After blocking with the Fc blocker reagent (BD Biosciences), the treated cells were incubated with three fluorescence-conjugated immunoglobulins, FITC-anti-AMPK (Catalog # AMPK2-FITC, Thermo Fisher Scientific), allophycocyanin–anti-HSL (custom conjugated Cat #PA5-64494, Thermo Fisher Scientific) FITC-IRF3 (Cat # CL48811312, Thermo Fisher Scientific), FITC-PPARγ (Cat # CL48860127, Thermo Fisher Scientific), FITC-CD86 (Cat # 53-0869-42, Thermo Fisher Scientific), and fluorescein isothiocyanate–anti-perilipin-1 (custom conjugated Cat #MA5-27861, Thermo Fisher Scientific) at 37 °C for two days. The stained cells were evaluated using a flow cytometer (FACScalibur, BD Biosciences), FlowJo 10.6.1 (BD Biosciences), and Prism 7 (GraphPad).

### 2.4. Conventional PCR

Total RNAs in the exposed cells under various conditions were isolated from the treated cells using the RiboEx reagent (GeneAll, Seoul, Republic of Korea), and cDNA was synthesized from the isolated RNA using Maxime RT PreMix (iNtRON, Seongnam, Republic of Korea). The cDNA was amplified with primers (Table 1) under the following cycling parameters: one minute at 95 °C, followed by 35 cycles of 35 s at 59 °C, and one minute at 72 °C. The amplified DNA was estimated using iBright FL1000 and iBright Analysis Software 4.0.0 (Invitrogen, Waltham, MA, USA).

### 2.5. Oil Red O Stain

The exposed cells were fixed with 4% paraformaldehyde (Sigma-Aldrich) for 20 min and washed with phosphate-buffered saline (Sigma-Aldrich). To detect neutral lipids and lipid droplets, cultured cells were stained with ORO (Sigma-Aldrich) prepared in isopropanol. Stained cells were observed under a fluorescence microscope (Eclipse Ts-2, Nikon, Shinagawa, Japan) and were analyzed with NIS-elements V5.11 (Nikon, Shinagawa, Japan).

### 2.6. Enzyme Immunosorbent Assay

The supernatants were isolated from the cells (fibroblasts and macrophages) exposed to various conditions to evaluate multiple cytokines. The isolated cytokines were assessed using reactive oxygen species (ROS) Fluorometric Assay Kit (Thermo Fisher Scientific), IL-10 (Interlekin-10) (Thermo Fisher Scientific), IL-6 (Interlekin-6) (Thermo Fisher Scientific), TNF-α (Tumor necrosis factor α) (Abcam, Cambridge, MA, USA), and IFN-β (Abcam) ELISA kits and a microplate reader (AMR-100; Allsheng, Hangzhou, China). To evaluate the expression of squalene synthase, adipose-derived stem cells (ASCs) were cultured under four conditions: CIE, PM10IE, SBEIE, and SBEIE + PM10IE. The cells were maintained in Dulbecco’s Modified Eagle Medium (DMEM, Invitrogen, Carlsbad, CA, USA) supplemented with Fetal Bovine Serum (FBS, Sigma-Aldrich, St. Louis, MO, USA) at 37 °C in a 5% CO_2_ incubator. After 24 h of incubation, the supernatants were collected and analyzed using the Squalene Synthase ELISA Kit (MyBioSource, Inc., San Diego, CA, USA).

Macrophages were exposed to four different conditions (CIE, PM10IE, SBEIE, and SBEIE + PM10IE), and the conditioned media obtained from these macrophages (CIECM; CIE-conditioned medium, PM10IECM; PM10IE-conditioned medium, SBEIECM; SBEIE-conditioned medium, SBEIECM + PM10IECM; SBEIE + PM10IE-conditioned medium) were collected. T lymphocytes (Jurkat cells, ATCC, Manassas, USA) were exposed to each conditioned medium for one day. The levels of IL-4 and IL-22 (R&D Systems, Minneapolis, USA) in each condition were measured using ELISA, with blank controls for each condition prepared from the respective conditioned media. The ELISA kits used for cytokine analysis had detection limits of 2 pg/mL (TNF-α), 1 pg/mL (IL-10), 0.5 pg/mL (IFN-β), 1 pg/mL (IL-4), 1 pg/mL (IL-6), 1 pg/mL (IL-22), and 0.3 ng/mL (squalene synthase). Standard curves were generated for each cytokine using serial dilutions, with R^2^ values ensuring accurate quantification.

### 2.7. Profiling of miRNAs in SBEIEs and Transfection of miRNA Candidates

Ebiogen Inc. (Seoul, Republic of Korea) sequenced the isolated and purified exosomes to analyze exosomal functions. AnAgilent 2100 Bio-analyzer and RNA 6000PicoChip (Agilent Technologies, Amstelveen, TheNetherlands) were used to evaluate RNA quality. RNA was quantified using a NanoDrop2000 spectrophotometer (Thermo Fisher Scientific, Waltham, MA, USA). Small RNA libraries were prepared and sequenced using an Agilent 2100 Bio-analyzer instrument for a high-sensitivity DNA assay (Agilent Technologies, Inc., Santa Clara, CA, USA), and the NextSeq500system was used for single-end 75 bp sequencing (Illumina, San Diego, CA, USA). To obtain an alignment file, the sequences were mapped using Bowtie 2 (CGE Risk, Lange Vijverberg, The Netherlands), and read counts were extracted from the alignment file using bedtools (v2.25.0) (GitHub, Inc., San Francisco, CA, USA) and R language (version 3.2.2) (R studio, Boston, MA, USA) to evaluate miRNA expression levels based the onhg38 genome. miRWalk 2.0 (Ruprecht-Karls-Universität Heidelberg, Medizinische FakultätMannheim, Germany) and ExDEGA v.2.0 (EbiogenInc., Seoul, Republic of Korea) were used for the miRNA target analysis. To evaluate transfection, fibroblasts were transfected with seven miRNAs (Table 2) using the Lipofectamine 2000 reagent (Invitrogen) with control siRNA oligonucleotide (negative) (Bioneer) and GFP-GAPDH siRNA (Bioneer) for one day. The supernatants from the transfected fibroblasts were evaluated for the expression levels of three markers (IL-6, MMP-9, and TNF-α; Thermo Fisher Scientific) using the ELISA method.

### 2.8. Evaluation of Clinical Effects by the Induced Exosomes

This study recruited 30 male and female participants in their 20 residing in Seoul, where exposure to air pollutants, such as fine dust and exhaust fumes, is severe. Participants were selected based on having three facial spots with the oil content exceeding 20% and moisture content below 40%, as measured using the SK-8 device (Feimiaomilei, Shanghai, China). All participants voluntarily provided their name, age, gender, email address, and phone number (P01-202411-01-047; approval date: 27 November 2024). To assess hypersensitivity, the test material (an essence containing 40 μg/mL of SBEIEs) was applied twice (once in the morning and once in the afternoon) on the same area of skin on the arm or hand. Participants with no adverse reactions (e.g., itching or rash) by the following day proceeded to the main study.

Measurements were conducted on days 0, 5, and 10. Evaluations included skin inflammation (porphyrin), moisture content, oil content, and skin imaging at the three identified facial spots using the JANUS 2 (PSI Co., Ltd., Seoul, Republic of Korea) and SK-8 devices. Participants also completed surveys assessing sebum reduction and satisfaction with the treatment.

The placebo group consisted of six participants randomly selected from 30 participants and were provided a formulation that did not contain exosomes. Considering potential dropouts, 24 participants were assigned to the exosome-containing formulation group. Ultimately, 20 participants completed the study successfully. The measurement results before and after product use were analyzed. A placebo-controlled group (six participants) was included under identical conditions for a robust efficacy assessment. The placebo was formulated with distilled water, glycerin (ELOGLYNTM, Seoul, Republic of Korea), and phosphate-buffered saline (PBS, pH 7.4; medical-grade PBS from Microgiene, Seoul, Republic of Korea). These substances excluded the active exosome component. Viscosity and color were adjusted to match the active formulation to ensure texture and sensory perception consistency. To investigate skin exposure to fine dust, the levels of heavy metals—Pb (Abcam), Cd (MyBioSource, San Diego, CA, USA), and As (Creative Diagnostics, Shirley, NY, USA)—were measured on the skin after outdoor exposure on days 0, 5, and 10. Samples were collected using sterile cotton swabs from three designated skin spots applicable for clinical measurements. Participants were single-blinded, meaning they were unaware of whether they received the SBEIE-containing formulation or the placebo. Study coordinators conducting measurements were also blinded to group allocation to ensure unbiased data collection.

### 2.9. Statistics

All in vitro (three independent experimental replications) and clinical (five measurements per individual per measurement day) experiments were analyzed using one-way analysis of variance (ANOVA) with post hoc analysis (Scheffe’s and Dunnett’s methods) using Prism 7 software (GraphPad). Before conducting the ANOVA, data normality was assessed using the Shapiro–Wilk test.

## 3. Results

### 3.1. Modulation of Lipogenic Cytokines by Two Materials

**Figure 1:**
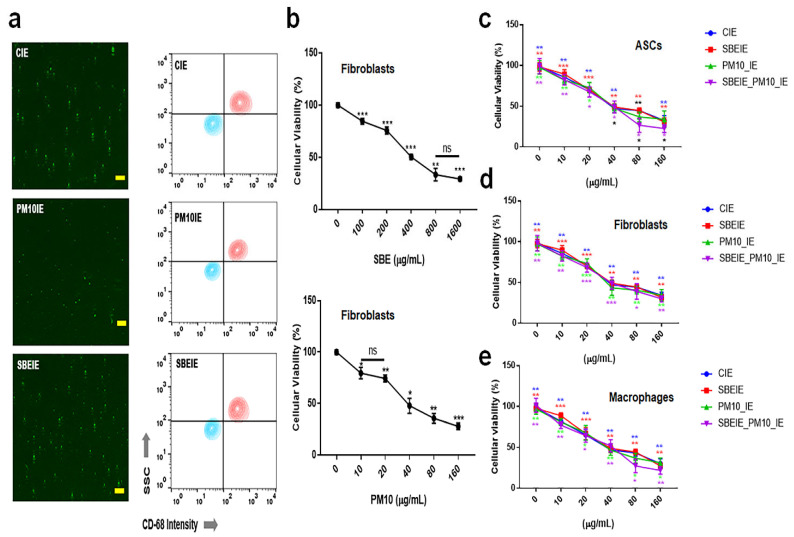
Purification of exosomes and the establishment of treatment dosages. (**a**) The images and the results of flow cytometry for the purification of the exosomes using FITC-CD68. (**b**) The cytotoxicity of *Scutellaria baicalensis* extract (SBE) and fine dust (PM10) to fibroblasts. (**c**) Cellular viability of induced exosomes (CIEs: control-induced exosomes, SBEIEs: SBE-induced exosomes, PM10IEs: fine dust-induced exosomes) in ASCs. (**d**,**e**) Cellular viability of induced exosomes in fibroblasts (**d**) and macrophages (**e**). ns: not significant (scale bars = 30 μm) (* *p* < 0.05, ** *p* < 0.01, *** *p* < 0.001).

**Figure 2:**
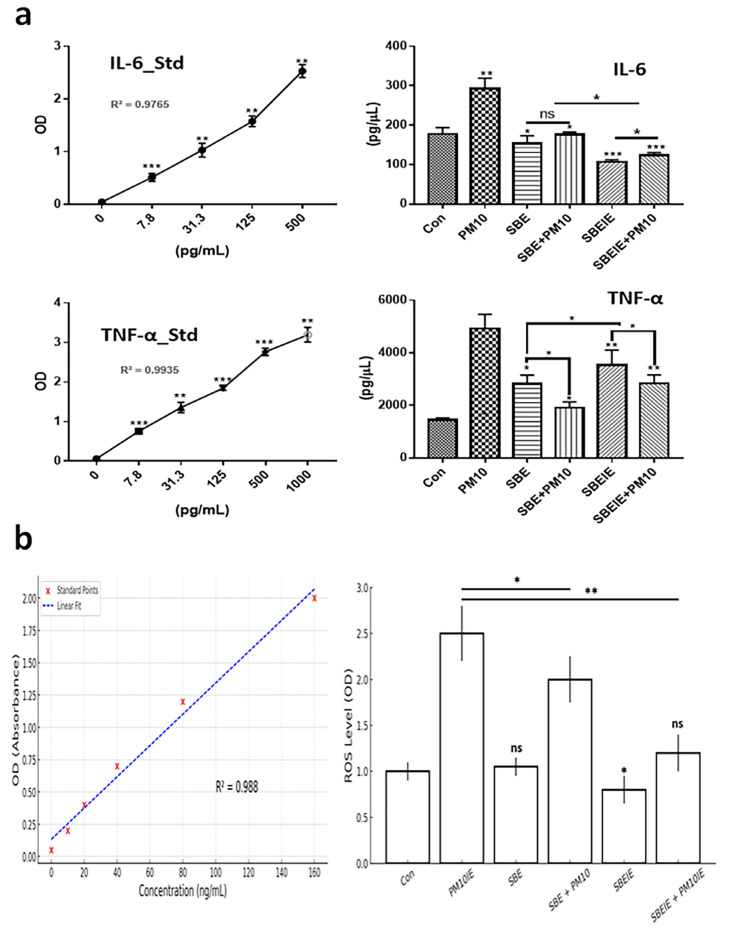
Expression levels of protective cytokines in fibroblasts under SBE and SBEIEs treatments against fine dust. (**a**) The bar graphs show the levels of secreted cytokines from fibroblasts under SBE and SBEIEs treatment; (**b**) ROS levels of fibroblasts exposed to various conditions. Con: control; PM10: fine dust; SBE: *Scutellaria baicalensis* extract; SBEIEs: SBE-induced exosomes; PM10IEs: fine dust-induced exosomes; OD: optical density; Std: standard, ns: not significant (* *p* < 0.05, ** *p* < 0.01, *** *p* < 0.001).

Exosomes induced under *Scutellaria baicalensis* extract (SBE) and PM10 were isolated from fibroblasts. After exosome purification and treatment dosage establishment (Figure 1), SBEs and SBEIEs were evaluated for their anti-lipogenic and ROS suppressive functions in fibroblasts (Figure 2). The cytotoxic activity of SBEIEs was comparable to that of CIEs and PM10IEs (Figure 1c–e). This similarity may be due to the intrinsic cellular response to exosome exposure, where fibroblasts, macrophages, and ASCs exhibit a baseline tolerance to exosomes regardless of their origin. While PM10IEs are expected to induce cytotoxicity due to fine dust components, the bioactive properties of SBEIEs might counteract these effects, stabilizing cell viability. The uniform purification and concentration of all exosomes (CIE, SBEIE, and PM10IE) during the experimental process may have contributed to the observed similarity in cytotoxicity. PM 10 activated lipogenic cytokine IL-6 in fibroblasts (Figure 2a). Under SBE and SBEIE treatments, fibroblasts exhibited a moderate increase in TNF-α, with more productive synthesis in SBEIEs than in the SBE (Figure 2a). However, exposure to PM10 induced an excessive rise in TNF-α in fibroblasts. SBEIEs were approximately 1.3 times more effective than the SBE (Figure 2a). Based on the results for ROS levels (Figure 2b), PM10 significantly amplifies ROS production in fibroblasts, whereas the SBE, particularly SBEIEs, effectively maintains ROS within the normal levels. SBEIE treatment induced TNF-α release, which may initially seem contradictory to its anti-inflammatory properties (Figure 2a). 

### 3.2. Modulation of Lipogenesis by SBEIEs in ASCs

**Figure 3:**
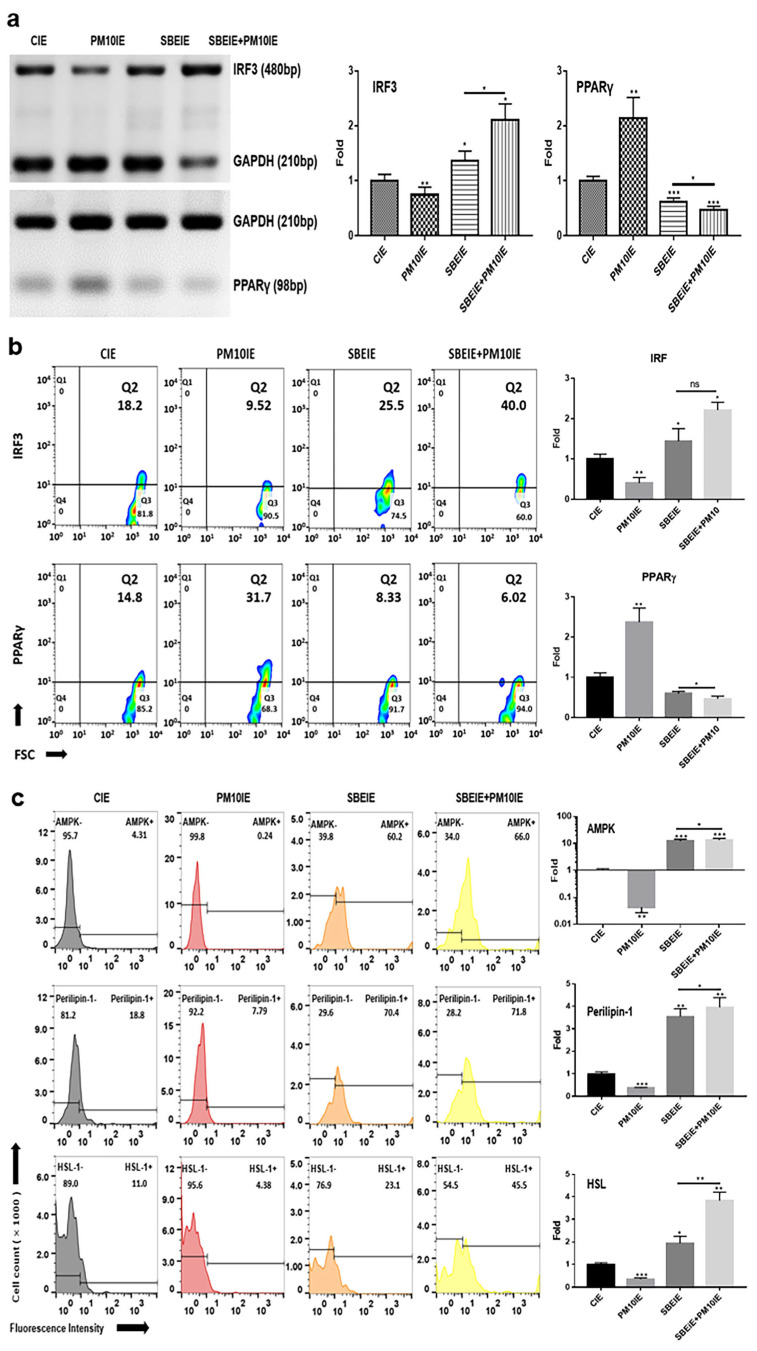
Alteration in lipogenesis by SBEIEs in ASCs following fine dust exposure. (**a**,**b**) The evaluation of the levels of lipolytic genes using conventional PCR and their expression levels, and (**c**) the expression levels of lipolytic markers using flow cytometry in ASCs (adipocyte-derived stem cells) under various conditions. HSL: hormone-sensitive lipase; CIEs: control-induced exosomes; PM10: fine dust; SBE: *Scutellaria baicalensis* extract; SBEIEs: SBE-induced exosomes; PM10IEs: fine dust-induced exosomes; OD: optical density; Std: standard; bp: base pairs; ns: not significant (* *p* < 0.05, ** *p* < 0.01, *** *p* < 0.001).

Based on the results of conventional PCR, SBEIEs and SBEIE + PM10IE activated the upregulation of IRF3 gene levels in ASCs (Figure 3). SBEIE + PM10IE was more effective than SBEIEs, increasing IRF3 levels by approximately 1.6 times (Figure 3a). This may be due to a compounding effect, where PM10-induced oxidative stress enhances the activation of IRF3-related pathways, further reinforcing SBEIEs’ effects. Similarly, the higher expression levels of perilipin-1 and HSL in the SBEIE + PM10IE condition suggest an augmented lipolytic response (Figure 3c). Given that PM10 exposure triggers metabolic stress, the combined presence of PM10IE and SBEIEs may drive an enhanced regulatory feedback mechanism, leading to a greater activation of lipid breakdown pathways.

**Figure 4:**
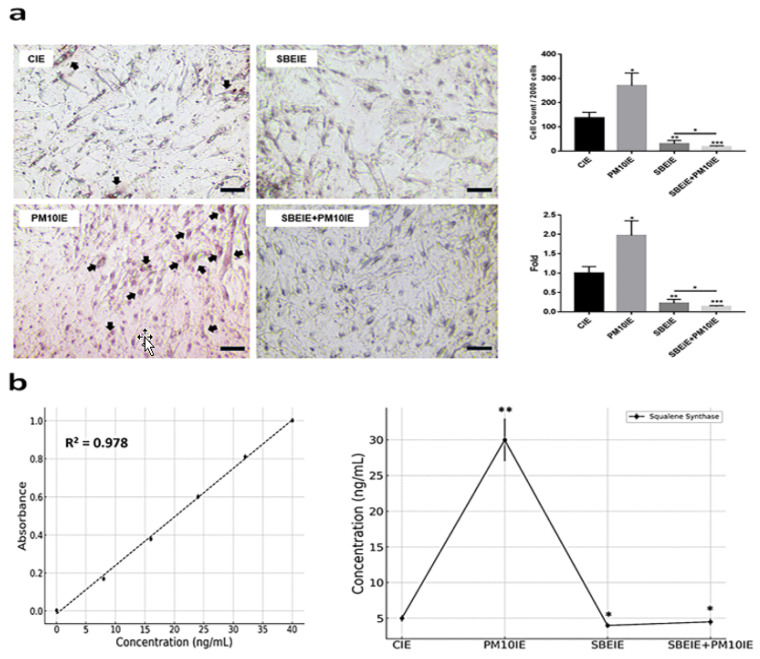
Modulation of lipogenesis by SBEIEs in ASCs upon fine dust exposure. (**a**) The exposed ASCs were stained using Oil Red O. (**b**) Expression levels of squalene synthase in ACSs exposed to the various conditions. The black arrows indicate positively stained cells. CIEs: control-induced exosomes; PM10: fine dust; SBE: *Scutellaria baicalensis* extract; SBEIEs: SBE-induced exosomes; PM10IEs: fine dust-induced exosomes (* *p* < 0.05, ** *p* < 0.01, *** *p* < 0.001) (scale bars = 20 μm).

In contrast to IRF3, the levels of PPARγ were significantly suppressed in ASCs exposed to both agents (Figure 3a). Similar to the PCR results (Figure 3a), the flow cytometry analysis of the two proteins showed that IRF3 expression increased under the SBEIE and SBEIE + PM10IE conditions compared to the control group. At the same time, PPARγ levels decreased (Figure 3b). Moreover, SBEIEs upregulated lipolytic markers, including AMPK, perilipin-1, and hormone-sensitive lipase (HSL), in ASCs (Figure 3b). Notably, under SBEIE + PM10IE treatment, perilipin-1 and HSL were intensely upregulated in ASCs (Figure 3b). Consistent with the results shown in Figure 3, the Oil Red O staining demonstrated that SBEIEs effectively activated lipolysis in ASCs (Figure 4a). The expression levels of squalene synthase, a specific marker of sebaceous gland-like cells, were measured under each condition, showing that PM10IEs significantly promoted the synthesis of squalene synthase. In contrast, SBEIEs reduced its synthesis by approximately 10% compared to the control (CIE). SBEIEs suppressed the increase in squalene synthase expression, even in the presence of PM10IE stimulation (Figure 4b).

### 3.3. Immune Modulation of SBEIEs

**Figure 5:**
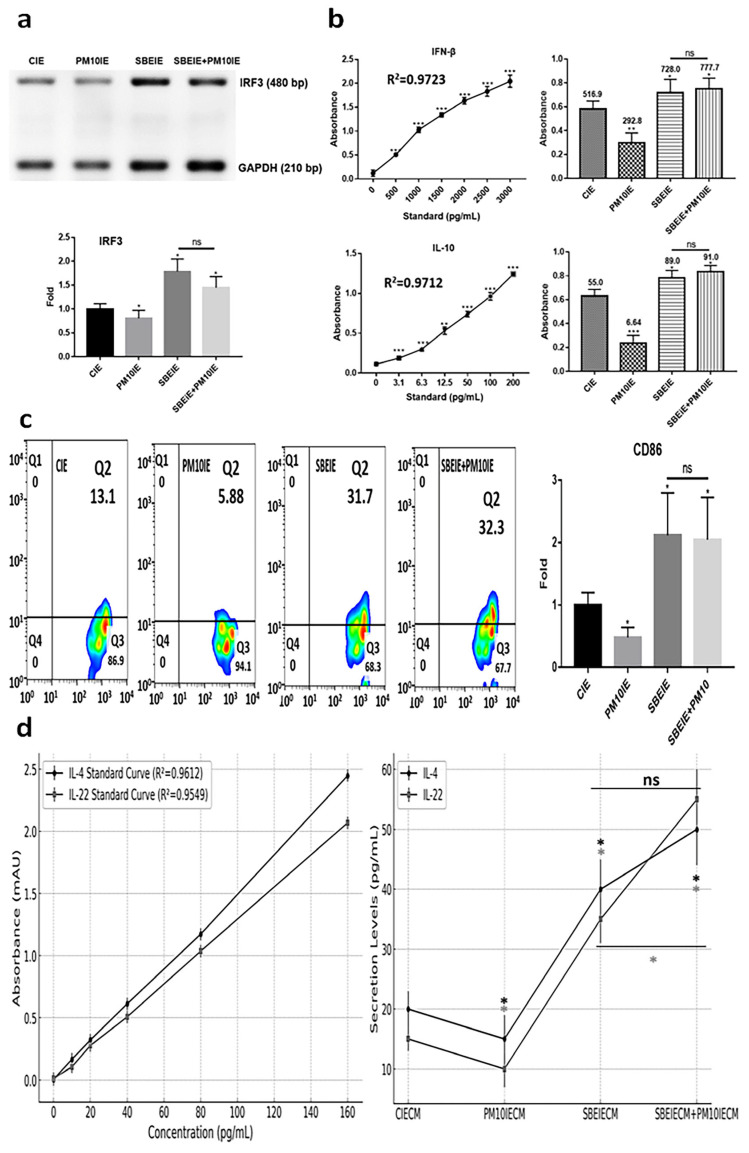
Immune modulation by SBEIEs in macrophages upon fine dust exposure. (**a**) Under various conditions, the levels of a key inflammation marker (IRF3) gene in macrophages. (**b**) The expression of anti-inflammatory cytokines (IFN-β and IL-10) in macrophages under various conditions. (**c**) Activation for polarization to M1 macrophages. (**d**) Expression levels of IL-4 and IL-22 in T cells under the four conditions: CIEs: control-induced exosomes; PM10: fine dust; SBE: *Scutellaria baicalensis* extract; SBEIEs: SBE-induced exosomes; PM10IEs: fine dust-induced exosomes; CIECM: CIE-conditioned medium; PM10IECM: PM10IE-conditioned medium; SBEIECM: SBEIE-conditioned medium; bp: base pairs; ns: not significant (* *p* < 0.05, ** *p* < 0.01, *** *p* < 0.001).

SBEIEs activated the upregulation of IRF3, IFN-β, and IL-10 in macrophages upon fine dust exposure (Figure 5a,b). Notably, unlike PM10, SBEIEs prevented the downregulation of these molecules induced by PM10 in macrophages (Figure 5a,b). Upon SBEIE treatment, IRF3 levels were upregulated approximately 2.1 times. Following the upregulation of IRF3, the levels of IFN-β and IL-10 were significantly increased in macrophages, regardless of fine dust exposure (Figure 5a,b). SBEIEs promoted the polarization of macrophages toward the M1 type (Figure 5c). T lymphocytes stimulated by macrophages exhibited increased secretion of IL-4, which inhibits sebaceous gland cell activation, and IL-22, which strengthens the skin barrier and suppresses sebaceous gland cell activity (Figure 5d). These cytokine levels increased by approximately 2 and 2.33 times compared to the control group under SBEIE stimulation. The secretion levels of both cytokines were elevated even in the presence of PM10IE exposure. TNF-α has context-dependent roles in immune regulation, and moderate increases can support lipolysis and immune modulation without exacerbating inflammation. The TNF-α increase observed in our study was accompanied by a concurrent rise in anti-inflammatory cytokines, such as IL-10 and IFN-β (Figure 5), suggesting that SBEIEs fine-tune the immune response rather than acting as a broad TNF-α suppressor. This indicates that SBEIEs may contribute to immune homeostasis and lipid regulation without promoting excessive inflammation.

### 3.4. miRNA Profiling and Their Characteristics in SBEIEs

**Figure 6:**
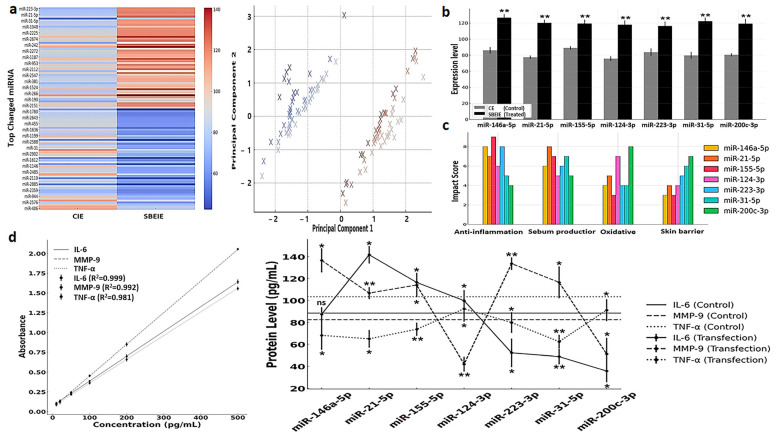
miRNA profiling and their characteristics in SBEIEs. (**a**) **Left**: Heatmap for top changed miRNAs in SBEIEs (*Scutellaria baicalensis* extract-induced exosomes) compared to CIEs (control-induced exosomes). **Right**: Principal component analysis (PCA) (red: upregulation, blue: downregulation). (**b**) Seven candidates in the top changed miRNAs for skin therapeutic biomaterial. (**c**) Four biochemical categories are associated with the seven candidates. (**d**). Standard curves and expression levels of three markers in fibroblasts transfected with each of the seven candidate miRNAs. ns: not significant (* *p* < 0.05, ** *p* < 0.01).

The profiling of 2500 miRNAs in SBEIEs identified seven miRNAs—miR-146a-5p, miR-21-5p, miR-155-5p, miR-124-3p, miR-223-3p, miR-31-5p, and miR-200c-3p—that are predicted to have therapeutic potential for seborrheic dermatitis (Figure 6a,b). Principal component analysis (PCA) was conducted to examine the clustering of miRNA expression profiles under CIE and SBEIE conditions (Figure 6a). The analysis revealed distinct groupings of differentially expressed miRNAs, suggesting shared regulatory mechanisms among clustered miRNAs (Figure 6a). The wide dispersion of certain miRNAs indicates diverse functional roles and differential responses to experimental conditions. The separation along principal components 1 and 2 highlights the significant variability in miRNA expression between the two conditions (Figure 6a). These findings indicate that specific miRNAs may act as key regulatory molecules in response to CIE and SBEIE conditions, emphasizing the need for further functional validation (Figure 6a). Biochemical analysis suggests these miRNAs exert effects across the four key categories, contributing to their expected efficacy in mitigating the condition (Figure 6c).

The ELISA analysis revealed that miRNA transfection significantly modulated IL-6, MMP-9, and TNF-α expression levels compared to the control condition, highlighting their therapeutic potential for seborrheic dermatitis. miR-146a-5p and miR-21-5p notably suppressed IL-6, a pro-inflammatory cytokine, indicating their role in mitigating chronic inflammation (Figure 6d). miR-155-5p and miR-124-3p effectively downregulated MMP-9, suggesting their contribution to extracellular matrix stabilization and skin barrier restoration (Figure 6d). Furthermore, miR-223-3p and miR-31-5p significantly reduced TNF-α levels, demonstrating their capacity to attenuate immune dysregulation and inflammation (Figure 6d). In contrast, the control samples exhibited higher levels of all three proteins, reflecting the untreated inflammatory state characteristic of seborrheic dermatitis. These findings collectively underscore the potential of these miRNAs to target key pathological mechanisms, including inflammation, skin barrier repair, and immune regulation, warranting further in vivo investigations and clinical studies (Figure 6d).

### 3.5. Clinical Effects of SBEIEs

**Figure 7:**
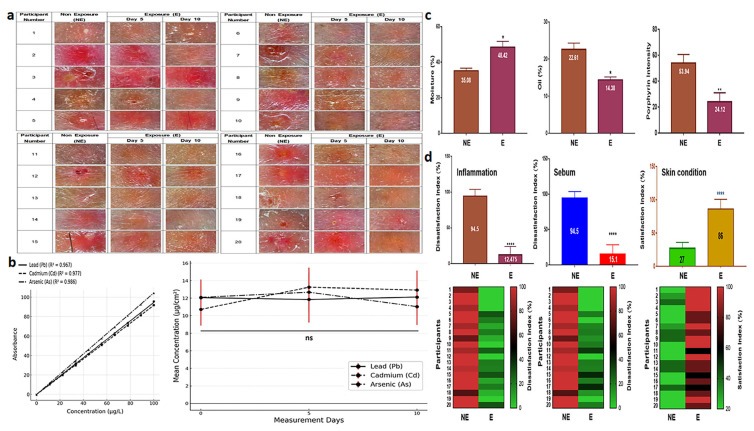
Therapeutic effects and survey results of SBEIEs on inflamed skin over ten days. (**a**) Images of inflamed skin exposed to an essence containing 40 μg/mL of SBEIEs for ten days were captured at the same dermal sites at three time points (days 0, 5, and 10). (**b**) Measurement values of skin heavy metals—Pb, Cd, and As—after outdoor exposure on days 0, 5, and 10. The analysis results represent 26 participants (including the placebo group) after excluding four dropouts. (**c**) Three parameters (moisture, oiliness, and porphyrin levels related to inflammation) were measured on the skin of 20 participants. The values were adjusted relative to those of a placebo-controlled group (six participants). NE: non-exposure; E: exposure to SBEIEs. (**d**) Three parameters (inflammation, sebum, and skin condition) were measured on the skin of 20 participants. The values were adjusted relative to those of a placebo-controlled group (six participants, S1). NE: non-exposure; E: exposure to SBEIEs; ns: not significant (* *p* < 0.05, ** *p* < 0.01, **** *p* < 0.0001).

SBEIEs demonstrated therapeutic effects both at the cellular level and in clinical trials. When an essence containing 40 μg/mL of SBEIEs was applied to inflamed facial skin, a significant improvement in the skin condition was observed within five days of treatment. By day 10, the skin condition had nearly returned to normal (Figure 7a). The results of measuring skin exposure to fine dust during outdoor activities over 10 days showed consistent levels on days 0, 5, and 10, indicating continuous skin exposure to fine dust (Figure 7b). The concentrations of Pb, Cd, and As were all approximately 12 µg/cm^2^ (Figure 7b). Measurements of three parameters (moisture, oiliness, and porphyrin intensity) revealed that SBEIEs increased moisture levels by approximately 21%, reduced oiliness by 1.6 times, and decreased inflammatory responses by 2.2 times compared to the pre-treatment values (Figure 7c). A skin satisfaction survey showed that improvement in inflammation increased by 7.6 times, sebum reduction by 6.3 times, and overall skin satisfaction by 3.2 times, consistent with the results shown in Figure 7d.

## 4. Discussion

The results of this study suggest that fine dust can promote lipogenesis in skin cells and adipose-derived stem cells and stimulate inflammatory responses, potentially leading to skin troubles. SBEIE biomaterials can inhibit skin issues by protecting against fine dust and suppressing lipid synthesis. Clinical results demonstrate a dramatic alleviation of skin problems.

The functions of SBEIEs in this study can be categorized into three main aspects. First, it regulates the secretion of cytokines that can control lipid synthesis in skin cells and adipose-derived stem cells. Second, SBEIEs can regulate lipid synthesis in adipose-derived stem cells. Third, it promotes inflammation suppression and immune cell differentiation in immune cells.

First, in fibroblasts, SBEIEs inhibited the synthesis of IL-6 and induced the synthesis of TNF-α (Figure 2). A moderate increase in TNF-α in skin cells can play several beneficial roles in maintaining skin health, supporting immune responses, and aiding tissue repair [36,37,38]. TNF-α significantly influences the differentiation process and lipid metabolism of ASCs, including upregulating AMPK, hormone-sensitive lipase (HSL), and perilipin-1 [10,39,40]. While low levels of TNF-α can promote metabolic activation, chronic or excessive exposure often impairs adipogenic differentiation and alters lipid synthesis pathways, contributing to inflammatory and metabolic disorders [41,42]. IL-6 is pivotal in developing and exacerbating skin troubles, including acne, psoriasis, and atopic dermatitis [43,44]. IL-6 affects the adipogenic differentiation and lipid metabolism of adipose-derived stem cells (ASCs), influencing their regenerative potential and metabolic activities, including lipid composition alteration, inflammation promotion, and lipogenesis enhancement in sebaceous glands [45]. The results suggest that SBEIEs regulate the secretion of these cytokines in skin cells to protect them from fine dust exposure. This regulation also affects lipid metabolism and differentiation in adipose-derived stem cells, playing a key role in preventing skin troubles. The results (Figure 2) highlight the critical role of *Scutellaria baicalensis* extract-induced exosomes (SBEIEs) in mitigating oxidative stress induced by fine dust exposure. Reactive oxygen species (ROS) play a crucial role in cellular homeostasis; however, excessive ROS production, particularly in response to environmental pollutants, such as PM10, leads to oxidative stress, inflammation, and lipid dysregulation in fibroblasts. Our results demonstrate that PM10 exposure significantly increases ROS levels in fibroblasts, whereas treatment with SBE and, more effectively, SBEIEs helped maintain ROS within normal levels (Figure 2b). These findings suggest that SBEIEs exert protective effects against fine dust-induced oxidative damage. A key regulatory mechanism underlying ROS modulation involves TNF-α and IL-6 inflammatory cytokines. PM10 exposure substantially increased TNF-α and IL-6 levels in fibroblasts (Figure 2a), contributing to ROS overproduction and amplifying oxidative stress. TNF-α activates nuclear factor-kappa B (NF-κB) and mitogen-activated protein kinase (MAPK) signaling pathways, leading to the upregulation of NADPH oxidase, a key enzyme involved in ROS generation [46]. Similarly, IL-6 plays a dual role in inflammation and metabolic regulation. Excessive IL-6 secretion promotes oxidative stress through JAK/STAT3 activation, enhancing mitochondrial ROS production [47]. The observed increase in ROS levels following PM10 exposure aligns with previous studies showing that airborne particulate matter exacerbates oxidative stress and inflammatory responses through cytokine-mediated pathways [48]. In contrast, SBEIE treatment led to a more controlled cytokine profile, characterized by a moderate increase in TNF-α and a suppression of IL-6, which contributed to maintaining ROS homeostasis. The ability of SBEIEs to regulate TNF-α levels suggests a protective role, as moderate TNF-α expression has been associated with adaptive immune responses and the activation of antioxidant pathways, such as Nrf2, which mitigates oxidative damage [49]. The reduction in IL-6 by SBEIEs is particularly significant, as IL-6 overexpression has been linked to oxidative stress-driven skin disorders, including acne and atopic dermatitis [50]. By modulating these inflammatory mediators, SBEIEs effectively counteract the oxidative burden of PM10.

Second, in the lipogenesis of ASCs, SBEIEs promoted the expression of markers associated with lipid breakdown in ASCs and demonstrated a protective effect against inhibiting lipid breakdown caused by fine dust exposure (Figure 3 and Figure 4). Lipid synthesis in ASCs plays a dual role in skin health, contributing to skin barrier maintenance and hydration under normal conditions while potentially exacerbating inflammatory conditions, such as acne, when overproduced. Understanding the mechanisms underlying ASC lipid synthesis and its regulation could pave the way for targeted therapies to address skin troubles [51,52]. Excess lipid accumulation in ASCs and sebocytes stimulates the release of pro-inflammatory cytokines, such as TNF-α and IL-6, aggravating skin inflammation, promoting sebaceous gland activity, and exacerbating acne through mTOR signaling activation [53,54].

Third, SBEIEs regulated the secretion of immunomodulatory cytokines by macrophages, which act as the orchestrators of the immune system [55]. IRF3 serves as a critical regulator of immune responses, offering potential benefits by reducing skin inflammation, enhancing tissue repair, the activation of stem cells, anti-aging, and supporting balanced lipid metabolism in ASCs [56,57]. IFN-β, a type I interferon, affects antimicrobial activity and barrier functions and promotes wound healing and skin barrier repair [58,59]. When secreted by macrophages, IFN-β activates the inhibition of lipid synthesis and promotion of lipolysis in ASCs [60,61]. IL-10 secretion by macrophages has protective effects on skin health by reducing inflammation, promoting wound healing, and maintaining sebaceous gland function [62,63,64]. It also plays a critical role in ASC differentiation and lipid synthesis, fostering an anti-inflammatory and metabolically balanced environment [65,66]. Based on these reports and the results (Figure 5), SBEIEs demonstrate that regulating the expression of IRF3 in macrophages alleviates skin troubles with IL-4 and IL-22 from activated T cells and promotes immune activation within adipose tissue by macrophages.

The profiling of 2500 miRNAs in SBEIEs identified seven key miRNAs with therapeutic potential for seborrheic dermatitis, reflecting their ability to modulate crucial biochemical pathways. These miRNAs were predicted to exert their effects across four categories: inflammation suppression, extracellular matrix stabilization, oxidative stress reduction, and immune homeostasis. For instance, miR-146a-5p and miR-21-5p have been extensively reported to inhibit NF-κB-mediated pro-inflammatory cytokine production, consistent with their observed suppression of IL-6 levels in this study [67]. miR-155-5p and miR-124-3p regulators of tissue remodeling [68] significantly downregulated MMP-9, indicating their contribution to skin barrier repair. Similarly, miR-223-3p and miR-31-5p reduced TNF-α, corroborating studies that highlight their role in immune modulation and inflammatory control [69]. Moderate increases in TNF-α promote lipolysis, whereas excessive secretion is known to induce inflammation [8].

The ELISA results demonstrate that these miRNAs modulate cytokine levels and address the pathological imbalance characteristic of seborrheic dermatitis. Control samples consistently exhibited elevated IL-6, MMP-9, and TNF-α, indicating an unregulated inflammatory microenvironment. The ability of miRNAs to simultaneously target multiple pathways underscores their potential as a multi-target therapeutic strategy, a critical advantage over conventional single-target therapies [70]. Their role in oxidative stress regulation aligns with the increased need for antioxidant mechanisms in inflammatory skin conditions. While these findings are promising, further in vivo validation and clinical trials are necessary to assess miRNA-based interventions’ safety, stability, and efficacy. Optimizing delivery systems, such as lipid nanoparticles or exosome-based vectors, could enhance the therapeutic applicability of these miRNAs [71].

The clinical application results (Figure 7) show that SBEIEs dramatically improve skin conditions by enhancing moisture retention, reducing oiliness, and alleviating inflammation. These improvements were consistent with the findings at the cellular level, demonstrating that this induced exosome has significant potential as a therapeutic material for treating skin diseases and represents a step closer to its application and industrialization.

Exosomes are promising bio-pharmaceutical materials due to their natural role as delivery vehicles for biomolecules [72]. They offer precise targeting, minimal immune rejection, and potential in regenerative medicine, skin disease treatments, and cancer therapy. Stem cell-derived exosomes, in particular, demonstrate significant regenerative and anti-inflammatory effects [73]. While our findings demonstrate the potential of SBEIEs in mitigating fine dust-induced skin inflammation and lipid dysregulation, certain limitations must be considered. The small sample size in this clinical study limits the generalizability, and long-term effects were not assessed. Exosome activity may vary based on donor cell conditions, which requires further standardization in future studies. Larger, long-term trials are necessary to validate the therapeutic application of SBEIEs.

## 5. Conclusions

This study highlights the therapeutic potential of SBEIEs and seven key miRNAs—miR-146a-5p, miR-21-5p, miR-155-5p, miR-124-3p, miR-223-3p, miR-31-5p, and miR-200c-3p—in addressing the critical mechanisms of seborrheic dermatitis and skin issues caused by fine dust exposure. SBEIEs protect skin cells by regulating ROS, increasing cytokine secretion, lipid metabolism, and immune responses, suppressing IL-6, modulating TNF-α levels, and promoting inflammation balance and tissue repair. It regulates lipid synthesis and stimulates lipolysis in adipose-derived stem cells, preventing excessive lipid accumulation and inflammation, while its effects on macrophage modulation enhance immune activation and anti-inflammatory outcomes. These findings, validated by ELISA and clinical results, demonstrate that SBEIEs and their miRNA-induced exosomes represent a promising biopharmaceutical approach for treating inflammatory skin diseases, repairing skin damage, and advancing industrial applications. Future studies are warranted to optimize SBEIEs’ delivery systems and validate their efficacy in vivo and clinical settings.

## Figures and Tables

**Table 1:** Primer design for conventional PCR.

Gene	Sequence (5′→3′)
*GAPDH* (Glyceraldehyde 3-phosphate dehydrogenase) *NM_002046.7*	Forward: GTGGTCTCCTCTGACTTCAACA Reverse: CTCTTCCTCTTGTGCTCTTGCT
*IRF3* (Interferon regulatory factor 3) *NM_001571.6*	Forward: GTGGGAGTTCGAGGTGACAG Reverse: CTACAATGAAGGGCCCCAGG
*PPARγ* (Peroxisome proliferator-activated receptor gamma) *NM_138712.3*	Forward: TCTGAGGACCACGACCTG Reverse: GCTGGTGCTGGTCTTGAG

**Table 2:** Sequences of seven miRNA candidates.

miRNA	Sequence
miR-146a-5p	UGAGAACUGAAUUCCAUGGGUU
miR-21-5p	UAGCUUAUCAGACUGAUGUUGA
miR-155-5p	UUAAUGCUAAUCGUGAUAGGGG
miR-124-3p	UUAAGGCACGCGGUGAAUGCCA
miR-223-3p	UGUCAGUUUGUCAAAUACCCCA
miR-31-5p	AGGCAAGAUGCUGGCAUAGCU
miR-200c-3p	UAAUACUGCCGGGUAAUGAUGGA

## Data Availability

Data are contained within the article.
